# Westminster Medical Society

**Published:** 1833-01

**Authors:** 


### HOSPITAL REPORTS.

### WESTMINSTER MEDICAL SOCIETY.

*A Case of Epilepsy, of twenty years' standing, detailed before the Westminster Medical Society, by* John Epps, m.d.

A. B., aged thirty-four, a married man, having had nine children, six of which are now living, has been afflicted with epileptic fits since the *eleventh* year of his age. These fits occurred, at first at intervals of a month, more lately of a fortnight, and still more frequently some time before coming under my care. He consulted me, when on a visit to Manchester, in the month of September of the year 1830. He had, as may be supposed, been under the care of several men of decided talent. One celebrated physician declared that the fits arose from a *too great determination of blood to the head.* Bleeding with leeches on the temples and the neck, and other means to diminish, according to the idea entertained, this determination, were adopted. Another celebrated practitioner considered the fits to be dependent *upon the presence of the tapeworm.* The Oleum Terebinthinæ retificatum was prescribed; but with no effect in relieving the disease. Other less noted practitioners treated the fits as dependent upon different causes, but without success. One eminent practitioner had the courage, the moral courage, to refuse to undertake the case; I say moral courage, because I consider it an act of high moral courage to refuse to undertake a case, when the refusal to undertake the same is founded upon the fact, that he, the practitioner, does not see the nature thereof.

Not having experienced any permanent benefit, and having tried so many plans, A. B. had given up all hopes of cure. This gentleman, however, knew a little of phrenology, and having heard of me as a phrenologist, as well as a physician, thought that I might, from the aid of phrenology, be better acquainted with the nature of his complaint than those physicians whom he had consulted, and who were either ignorant of, or unbelievers in, the science; and as, in addition, the old axiom is very generally received, even beyond the sacred boundaries of our brethren's studies, that "a knowledge of the disease is half the cure," A. B. imagined that, perchance, some means might be discovered for the cure of his complaint, to him peculiarly troublesome, his pursuits being *intellectual* in their character. On these accounts he consulted me.

After examining minutely into the case, I told him that, though *many* symptoms militated against the hope of recovery, *more* symptoms seemed to justify that hope; and that, such being my opinion, I would undertake the case. His friends, and he himself, looked upon this as the last trial to be made.

I may, advantageously perhaps, briefly state the condition of the patient. A severe dyspepsia affected him; excessive nervousness; frequent and severe pains in the head, attended with soreness of the muscles of the neck. The fits occurring as before stated.

It appeared, therefore, that there were two objects in view, namely, to relieve the dyspepsia, and to remove the cerebellic affection.

To relieve the *dyspepsia*, I ordered the following mixture: ℞. Acidi Hydrocyanici, ad formulam Domini Scheele, gtt. xxx.; Sulphatis Quininæ, ʒss,; Sulphuris Præcipitati, ʒiss.; Confectionis Aromaticæ, gr. xv.; Aquæ distillatæ, f℥iij. He was ordered to keep the mixture carefully excluded from the air and the light, and of it to take a teaspoonful three times a day.

To relieve the *cerebellic* affection, (for I am forced to believe that epilepsy is connected with a diseased state of the cerebellum,) I prescribed the following liniment, to be rubbed diligently down the spine every night: ℞. Linimenti Saponis, f℥i.; Linimenti Camphoræ comp. f℥ss.; Tincturæ Lyttæ, fʒiv.; Ol. Succini. rectificati, fʒvi.

A. B. began attending to my regulations on Sunday, September 26th, 1830. During the first week, he had great pain and weakness in the back, (occasioned, A. B. thought, by the rubbing); also violent stitches and sudden pains through the day, darting from the spine. This latter circumstance seems to be worthy of remark. It is right also to notice, that A. B. had experienced an attack of the epileptic fit a few days before I saw him.

During this first week, already noticed, the *nervous agitation was excessive*, and on Monday, October 4th, he, in expressing the feeling in his head, compared it to "*a whirlpool*:" indeed, so peculiar was the feeling, that, though, as may be conceived, he had experienced an almost infinite variety of feelings, he asserts he never experienced so peculiar a feeling before. "Since that time," he states in his first communication to me, "I have felt much better;" and, "since Saturday," that is, the Saturday following the Monday specified, "I have felt so light and free from pain that I could fancy springs in my shoes when I am moving."

The occurrence of this peculiar feeling on the 4th of October may, in part, be referred to the circumstance of its being about the time of the *bihebdomadal* attack.

The rubbing on the back produced considerable eruption, which, on account of the itching connected with it, was extremely troublesome; a circumstance I did not regret.

A. B. found that the effects described, as well as others not noticed, were so different from those produced by the modes of treatment previously adopted, that he was much encouraged to persevere.

The dyspeptic mixture and the friction were used for a fortnight; the patient, in addition, bathing his feet alternate nights in hot water.

Perceiving the effect I expected was in progress of obtainment, I wrote to desire him to desist from the use of the liniment for a week, and, at the expiration of that period, to have recourse to it again three times a week, for three successive weeks. The mixture was again ordered; but, as the dyspepsia was much relieved, only to be taken twice a day.

Believing that the disease was within the power of remedial means, and also that the diseased condition was a want of power in the nervous fibres, constituting the cerebellum, (and I may remark here, that I intended to show to the Society a preparation, exhibiting the laminated structure of that organ, but being unable to reach home before coming to the Society, I am not able,) I ordered, in addition to the means already noticed, a liniment, consisting of the alcoholic extract of Nux Vomica, dissolved in rectified spirit, to be rubbed upon the back of the head for eight days. The patient commenced the use of this liniment on the 17th of October. Three days after the commencement of this friction, the nervous agitation, before described, again occurred very severely; a recurrence, it will be observed, occurring about the bi-hebdomadal period. From this date to November the 6th, the patient felt little of it.

It may be proper to notice, that, during the week when the rubbing of the back was suspended, the shooting pains from the spine, before described, again occurred, and were troublesome; being removed, however, when friction was had recourse to. In addition hereto, it is necessary to notice that a heat and a pain at the back of the head were much relieved by the friction on that part.

Up to this period the patient had been free from fits; a time of freedom much longer than usual, as he himself observed.

In addition to these means, I recommended A. B., with the view to the tonic effect, to wash the chest and the neck every morning with cold water, and to rub dry with a rough, well-dried cloth. He was requested to disuse the head liniment for fourteen days, at the expiration of which period he was ordered to use it for seven days.

During the interval from the 28th of October to November 6th, the patient had been troubled with the formation of a boil just below, as he states in his letter, " what you call the organ of amativeness." This boil I considered as very useful, and gave me an opportunity of still further encouraging him; for, it must be well known that the formation of boils is very often a very favorable index.

In reply to his letter of November 6th, I desired A. B. to take the following powder every morning: ℞. Sulphatis Quininæ, gr. iij.; Pulv. Cinnamomi, gr. i.; and to rub the back every other week. This recommendation was attended to for eight weeks: and during this period, the liniment for the head was used twice for seven successive nights.

Between this and the 1st of January, A. B. was troubled with a severe cold, and with an attack of English cholera; the latter of which I am inclined to ascribe to the quinine, which acts peculiarly on the liver.

The patient, in his letter of January the 1st, expresses great gratitude that he has begun the new year under such favorable circumstances, not having had an attack of the fits since I prescribed for him. To obviate the injurious effect on the liver, arising from the too long continuance of quinine, I ordered a Seidlitz powder, with a teaspoonful of the best Cogniac brandy and water, to be taken twice a week; and the head to be rubbed with salt water; the back being rubbed with the liniment once a month for three nights successively.

It is a well-known fact, that, in the spring of the year, diseases, which have been lying dormant, often develop themselves, either in their former characters or under their modifications. A. B., in a letter, dated March 4, states " for the last two or three months, I have had a periodical attack every fortnight of vomiting in the night. It causes me to spring out of bed before it awakens me. The vomiting continues most violent for some time, and little, save a frothy matter, comes off the stomach." On one occasion, the patient awoke before the vomiting came on, and the sensation experienced was that of a round substance at the bottom of the throat, which went down gradually till it reached the stomach. The vomiting then began. After the vomiting ceased, this round substance seemed to pass into the bowels, and died away.

This curious modification of the disease attracted the attention of A. B. so much the more, because he had not, previously to this, been subject to vomiting.

Another evidence of the change assumed by the disease was that for three weeks, previous to the 4th of March, A. B. experienced a violent burning heat exactly at the crown of the head, occupying a hand's breadth, striking down the back of the head, and attended with a sensation of great weight on each side of the lower part of the brain. This was attended with great mental confusion; and so severe was this pain, that the least motion of the head to either side caused a sensation similar to that which usually occurred after an attack of the fits, namely, that of the muscles of the neck being strained.

Being convinced that the vomiting was periodic and spasmodic, I

might say hysterical in its character, I ordered the liquor arsenicalis in the dose of three drops three times a day, and the following pills: ℞. Assafœtidæ lachrymarum, gr. xii.; Sulphatis Quininæ, gr. viij. M. bene ut fiat pil. vi. duæ h. s. s., three nights before the expected attack, and to be taken for three successive nights. At the same time I forwarded to him, with the view of keeping the bowels regular, and also to restore the liver to a healthy condition, a box of pills, prepared with the compound rhubarb pill and the dandelion extract, as made according to Mr. Houlton's formula by Smith, of Brown street, Edgeware road. I am thus particular in specifying, because I know but that one place in London where good Extractum Taraxaci can be obtained.

The means prescribed were attended with success. Still, however, between this period and the latter end of November, 1831, A. B. was troubled at intervals of two months with the suffocating feeling, the retchings and vomitings; the suffocating feelings continuing sometimes day and night. He wrote to me requesting to know whether I thought fit to recommend any other means, as, in his opinion, the phenomena described originated in the removal of the fits. This idea I appreciated; and, looking, as I did, on these symptoms as indicative of the attempts of the vital powers to relieve the system from the affection of the brain, I considered it my duty to recommend the introduction of an issue, as a means by which the diseased action might be effectually removed from the brain.

On March the 23d, 1832, I received a letter from A. B., in which he stated that the insertion of the issue had been attended with the desired effect.

In the summer of 1832, A. B. went a journey, which still further promoted his cure. He is now entirely free from fits.

It will be observed that I did not order the friction on the back of the head until I had tried and used the liniment on the spine. The reason is, because I find the friction on the spine relieves cerebral congestion; and, this being removed, I then desired to apply the tonic application, the solution of the alcoholic extract of nux vomica.

Such is the case of epilepsy to which the Society has been kind enough to listen.

Before concluding, it may be proper to make one or two observations. The first is, that length of time is not to be our index as to the curability of epilepsy: this patient had been afflicted with the disease for twenty years. The second is, that marriage did not remove the epilepsy, as it often does in persons of a full habit; the seminal evacuations relieving the cerebellum. The third is, that in cases of epilepsy attended with severe dyspeptic symptoms, a cure is more likely than in those cases not so attended. The fourth is, the peculiar modification the disease assumed. The fifth is, the benefit of the issue.

